# Asparaginyl-tRNA synthetase (*NARS1*) variants implicated in dominant neurological phenotypes display dominant-negative properties

**DOI:** 10.1016/j.xhgg.2025.100519

**Published:** 2025-09-18

**Authors:** Sheila M. Peeples, Keyana Blake, Brendan L.M. Sutton, Marina Konyukh, Stephan Züchner, Tanya Stojkovic, Jonathan Baets, Anthony Antonellis

**Affiliations:** 1 Department of Human Genetics, University of Michigan Medical School, Ann Arbor, MI 48109, USA; 2 Neuroscience Graduate Program, University of Michigan, Ann Arbor, MI 48109, USA; 3 AP-HP, Departement de Genetique Medicale, Hopital Henri Mondor, 94010 Créteil, France; 4 Dr. John T. Macdonald Foundation, John P. Hussman Institute for Human Genomics, University of Miami, Miami, FL 33136, USA; 5 Centre de Référence des Maladies Neuromusculaires Nord/Est/Ile de France, Institut de Myologie, Hôpital Pitié-Salpêtrière, Assistance Publique des Hôpitaux de Paris, Paris, France; 6 Translational Neurosciences, Faculty of Medicine and Health Sciences, University of Antwerp, 2610 Wilrijk, Belgium; 7 Laboratory of Neuromuscular Pathology, Institute Born-Bunge, University of Antwerp, 2610 Wilrijk, Belgium; 8 Neuromuscular Reference Centre, Department of Neurology, Antwerp University Hospital, 2650 Edegem, Belgium; 9 Department of Neurology, University of Michigan Medical School, Ann Arbor, MI 48109, USA

**Keywords:** peripheral neuropathy, aminoacyl-tRNA synthetase, dominant-negative effects, Mendelian disease

## Abstract

Aminoacyl-tRNA synthetases (ARSs) are essential, ubiquitously expressed enzymes that ligate amino acids to cognate tRNAs in the cytoplasm and mitochondria. To date, seven dimeric ARS enzymes have been implicated in dominant inherited neuropathy, suggesting that tRNA charging—exacerbated by a dominant-negative effect—is a component of the peripheral nervous system (PNS) phenotype. Interestingly, heterozygosity for missense and protein-truncating variants in the gene encoding dimeric, cytoplasmic asparaginyl-tRNA synthetase (*NARS1*) have been associated with distinct clinical phenotypes where patients present with either an isolated PNS neuropathy or with a complex phenotype that includes both PNS and central nervous system (CNS) features. Thus, *NARS1* variants are associated with a spectrum of dominant neurological diseases. Here, we test pathogenic *NARS1* variants for dominant-negative properties to determine if this mechanism is a common feature of ARS-related dominant neurological disease. Furthermore, we assess if variable dominant-negative effects explain the observed clinical heterogeneity. We performed yeast complementation assays to test *NARS1* variants in isolation, which revealed loss-of-function effects. To test for dominant-negative properties, we co-expressed mutant human *NARS1* with wild-type human *NARS1*. These studies revealed that *NARS1* variants interact with the wild-type subunit and that the majority of variants repress the ability of the wild-type allele to support cellular growth, consistent with a dominant-negative effect. Furthermore, our data suggest that *NARS1* variants associated with CNS and PNS phenotypes have a more severe dominant-negative effect compared with those associated with an isolated PNS phenotype.

## Teaser

Mutations in seven aminoacyl-tRNA synthetase cause dominant neurological disease with varying severity. To date, a unifying mechanism has not been identified for these related phenotypes. This study demonstrates that dominant *NARS1* variants have dominant-negative (DN) properties. Furthermore, the study provides evidence that variable DN effects explain the observed clinical heterogeneity.

## Introduction

**Figure 1:**
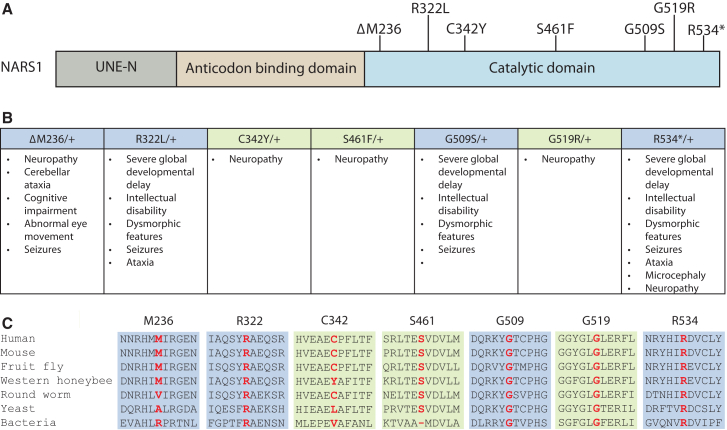
Localization and conservation of *NARS1* variants (A) NARS1 functional domains are indicated in gray (unique N-terminal extension), beige (anticodon binding domain), and blue (catalytic domain). The position of each *NARS1* variant is displayed across the top. (B) Phenotypes associated with each *NARS1* variant are provided. Green indicates a PNS-only phenotype while blue indicates PNS and CNS features. (C) The conservation of affected amino acid residues. The position of each variant is indicated alongside neighboring NARS1 amino acid residues from evolutionarily diverse species. The position of the affected residue is indicated by bold, red text. Green shading indicates a PNS-only phenotype while blue shading indicates PNS and CNS features.

Aminoacyl-tRNA synthetases (ARS) are ubiquitously expressed, essential enzymes that charge tRNA to cognate amino acids.^1^ To date, mutations in seven ARS loci have been implicated in dominant axonal peripheral neuropathy: glycyl-(*GARS1*; MIM: 600287),^2^ alanyl-(*AARS1*; MIM: 601065),^3^ tyrosyl-(*YARS1*; MIM: 603623),^4^ histidyl-(*HARS1*; MIM: 142810),^5^ tryptophanyl-(*WARS1*; MIM: 191050),^6^ seryl-(*SARS1*; MIM: 607529),^7^ and asparaginyl-(*NARS1*; MIM: 108410)^8^^,^^9^ tRNA synthetase. Interestingly, pathogenic variants in *NARS1* (Figure 1A), which is responsible for charging tRNA with asparagine in the cytoplasm, have also been associated with a dominant neurological disorder that affects both the central and peripheral nervous system (PNS). This *NARS1*-associate phenotypic spectrum includes global developmental delay, intellectual disability, microcephaly, dysmorphic features, seizures, ataxia, and peripheral neuropathy (Figure 1B).^8^^,^^10^ All reported *NARS1* variants characterized in this study are located in the catalytic domain, affect highly conserved residues, and are either not detected or detected at low frequencies in gnomAD (Figures 1A–1C).^8^^,^^9^^,^^10^

To date, pathogenic ARS variants associated with dominant neurological disease include missense alleles, small in-frame deletions,^7^^,^^8^^,^^9^^,^^10^^,^^11^ and one reported protein-truncating variant.^10^ Pathogenic variants result in complete or partial loss-of-function effects in functional assays.^8^^,^^9^^,^^10^^,^^11^^,^^12^ Haploinsufficiency is unlikely to be the underlying mechanism leading to disease, as null ARS alleles are found in the heterozygous state in unaffected individuals^13^ and null alleles have not been identified in individuals who present with dominant disease. Additionally, mice heterozygous for a *Gars1* null allele do not exhibit a neuropathy phenotype.^14^ Previous studies have suggested a toxic gain-of-function effect,^11^ where pathogenic ARS alleles lead to changes in protein structure, exposing protein interfaces and allowing novel protein-protein interactions that disrupt pathways involved in neuronal function.^15^^,^^16^^,^^17^^,^^18^^,^^19^^,^^20^ However, such effects do not explain the pathogenicity of all neuropathy-associated ARS alleles. For example, while some GARS1 mutant proteins have been shown to aberrantly interact with neuropilin-1, this novel interaction does not apply to all GARS1 mutants. Notably, studies have shown that the most severe *GARS1* allele associated with an infantile-onset spinal muscular atrophy phenotype, ΔETAQ, does not interact with neuropilin-1, suggesting that other pathogenic mechanisms are involved.^21^

Recent studies have demonstrated that expression of neuropathy-associated variants leads to an inhibition of global protein translation *in vitro*^6^^,^^22^^,^^23^ and *in vivo*,^24^^,^^25^ an increase in elongation initiation factor 2 alpha phosphorylation,^22^^,^^23^^,^^24^ and activation of the integrated stress response.^22^^,^^23^^,^^24^ Furthermore, *GARS1* mutations have been shown to sequester tRNA and hinder the ability to release tRNA.^23^^,^^26^ Combined, the above studies are consistent with a pathogenic mechanism where ARS variants affect protein translation through a decrease in the enzyme’s ability to charge or release tRNA, ultimately leading to disease.

We recently demonstrated that pathogenic alanyl-tRNA synthetase (*AARS1*) alleles exhibit dominant-negative effects in a humanized yeast model.^27^ A dominant-negative mechanism is relevant for gene products that form dimers and prerequisites for this mechanism include: (1) stable expression of the mutant gene product, (2) reduced or ablated function of the mutant gene product, (3) retained interactions between the mutant and wild-type gene products, and (4) repression of the function of the wild-type gene product by the mutant gene product.^11^ With regard to ARS-related disease, a dominant-negative mechanism is supported by the fact that all seven implicated ARS enzymes function as obligate homodimers and data showing that: (1) the majority of pathogenic ARS variants lead to reduced gene function,^6^^,^^7^^,^^21^^,^^28^^,^^29^^,^^30^^,^^31^^,^^32^ (2) pathogenic variants do not lead to reduced protein expression,^21^^,^^33^^,^^34^ (3) pathogenic variants do not ablate interactions with the wild-type ARS,^4^^,^^6^^,^^7^^,^^22^^,^^32^^,^^35^ and (4) mutant AARS1 represses the function of wild-type AARS1 in a dimer-dependent manner in a humanized yeast assay.^27^ Please note that, while *in vitro* assays have shown that AARS1 can function as a monomer, we have shown that AARS1 must dimerize to support cellular growth in an *in vivo* yeast model.^27^ Importantly, the other six neuropathy-associated ARS loci (including *NARS1*) have not been tested for dominant-negative properties; pursuing these studies is essential for evaluating dominant-negative effects as a common mechanism across all seven implicated ARS loci.

Here, we describe a *NARS1*-specific humanized yeast assay and we leverage this model to demonstrate that *NARS1* variants implicated in dominant neurological disease have both loss-of-function and dominant-negative properties. Furthermore, we provide insight into genotype-phenotype relationships at *NARS1*: a more severe dominant-negative effect was observed for variants associated with both central nervous system (CNS) and PNS phenotypes compared with variants associated with isolated PNS phenotypes. Our efforts have important implications for defining the mechanism of dominant ARS-related diseases, assessing the pathogenicity of newly identified *NARS1* variants, and developing therapies for patients with *NARS1*-related clinical phenotypes.

## Material and methods

### Conservation and population frequency

The frequency of each *NARS1* variant in the general population was assessed by interrogating the gnomAD database.^13^^,^^36^ To evaluate the conservation of each variant, NARS1 protein orthologs from the indicated species (Figure 1C) were aligned using Clustal Omega.^37^ The accession numbers for NARS1 protein sequences used in this analysis were as follows: human (*Homo sapiens*, NP_004530.1), mouse (*Mus musculus*, NP_001136422.2), fruit fly (*Drosophila melanogaster*, NP_609948.1), western honeybee (*Apis mellifera*, XP_623490.2), roundworm (*Caenorhabditis elegans*, NP_001021405.1), yeast (*Saccharomyces cerevisiae*, NP_011883.1), bacteria (*Escherichia coli*, NP_309040.1).

### Yeast vector constructs and growth assays

All yeast assays were performed using the haploid ptetO7-*DED81* strain from the Yeast Tet-Promoters Hughes Collection (Horizon Discovery), which harbors a doxycycline-repressible promoter at the *DED81* locus (the yeast ortholog of *NARS1*). *NARS1* expression constructs were generated using Gateway cloning technology (Invitrogen). The wild-type human *NARS1* open reading frame was amplified from human cDNA and confirmed via Sanger sequencing. The Gateway cloning (Invitrogen) BP reaction was performed to recombine wild-type *NARS1* into pDONR221. *NARS1* variants were generated using site-directed mutagenesis (Agilent QuikChange II XL Site-Directed Mutagenesis Kit); primers are available upon request. All resulting clones were validated via Sanger sequencing and LR-cloned (Invitrogen) to recombine the wild-type or mutant *NARS1* open reading frame into the Gateway-compatible pAG425GAL-ccdB vector (Addgene, no. 14153). The pAG425 vector contains a *GAL1* promoter to direct the expression of *NARS1* in a galactose-inducible fashion, a 2-μm origin of replication to yield a high-copy vector number per cell, and a *LEU2* auxotrophic marker.

To evaluate the function of *NARS1* variants, a p413 vector (ATCC, no. 87370) with no *NARS1* insert (“Empty”) was transformed into the ptetO7-*DED81* strain. The p413 vector contains an *ADH1* promoter to constitutively express the target gene, a centromeric origin of replication to yield a low-copy vector number per cell, and a *HIS3* auxotrophic marker. A second transformation was then performed to introduce a pAG425 vector harboring wild-type or mutant *NARS1*. Transformations were plated on media lacking histidine and leucine to select for the presence of both vectors and grown at 30°C for 3 days. Colonies were picked into 2 mL of media lacking histidine and leucine in a 14-mL round-bottom canonical tube until saturated for 2 days at 30°C, shaking at 275 rpm. Yeast samples were then diluted 1:10, 1:100, and 1:1,000. Ten microliters of undiluted or diluted samples were spotted on plates lacking histidine and leucine and containing glucose, 2% galactose/1% raffinose (Formedium) or 2% galactose/1% raffinose with 10 μg/mL doxycycline (Fisher Scientific). Yeast samples were imaged after 3 and 5 days of growth at 30°C.

To co-express mutant and wild-type *NARS1* in yeast, the LR Gateway reaction was performed to recombine wild-type *NARS1* into the previously described Gateway-compatible p413 vector.^27^ This construct was transformed into the ptetO7-*DED81* strain, followed by transformation of pAG425 vectors to express wild-type or mutant *NARS1*. To co-express mutant *NARS1* from a low-copy vector, the ptetO7-*DED81* strain containing wild-type *NARS1* on p413 was transformed with pAG415 (a low-copy, centromere-bearing vector) harboring wild-type or mutant *NARS1*. Subsequent selection and spotting were performed as described above. Yeast samples were imaged after 3 days of growth at 30°C. Yeast spots were quantified by obtaining the mean gray values using ImageJ.^38^ At least five transformations were performed and one to two colonies per transformation were picked.

For growth curve analysis, yeast co-expressing mutant and wild-type *NARS1* were grown in 4 mL of media lacking leucine and histidine to select for the presence of both vectors and grown at 30°C for 2 days, shaking at 275 rpm. The OD_600_ for saturated cultures was measured and diluted to a starting OD_600_ of 0.1 in liquid media lacking leucine and histidine and containing 2% galactose/1% raffinose with 200 μg/mL doxycycline. Diluted cultures were aliquoted into a 96-well clear flat-bottom non-treated plate (Corning, no. 3370), covered with an optical overlay and the OD_600_ was measured every hour for 48 h using a BioTek Epoch 2 Microplate Spectrophotometer.

### Yeast protein isolation

Protein isolation from yeast cultures was performed as previously described.^27^ Briefly, wild-type or mutant *NARS1* expression constructs were transformed into ptetO7-DED81 and grown on media lacking leucine. One colony was picked into 3 mL of media containing 2% galactose/1% raffinose and lacking leucine and doxycycline, and grown for 2 days at 30°C, shaking at 275 rpm. Yeast cultures were centrifuged at 1,000 × *g* for 10 min, washed once with water, transferred to a 1.5-mL Eppendorf tube, and then centrifuged at 15,000 rpm for 1 min. The supernatant was discarded and the pellet was stored at −80°C. The pellet was thawed in 200 μL of yeast lysis buffer (50 mM Na-HEPES [pH 7.5], 100 mM NaOAc, 1 mM EDTA, 1 mM EGTA, 5 mM MgOAc, 5% glycerol, 0.25% NP-40, and 3 mM DTT) with 1× Halt Protease Inhibitor Cocktail (Thermo Fisher Scientific). Approximately 100 μL of 0.5-mm cold glass beads (Biospec Products) were added to each sample. The samples were vortexed at 4°C for 3 min, followed by 2 min of resting on ice, and another 3 min of vortexing at 4°C. To remove the lysate from the beads, a 26-gauge needle (BD PrecisionGlide) was used to make a hole in the bottom of the 1.5-mL tube and then immediately inserted into a 14-mL round-bottom conical tube. Lysates were centrifuged at 200 × *g* at 4°C for 5 min. The lysates were collected from the bottom of the conical tube, transferred to a 1.5-mL Eppendorf tube, and centrifuged at 13,200 rpm for 10 min at 4°C. Supernatants were collected, and protein concentrations were measured using the Thermo Scientific Pierce BCA Protein Assay Kit.

### Western blot analyses

Western blots were performed to assess the levels of human NARS1 in yeast. To prepare each sample, 50 μg of protein was combined with 1× Novex Tris-glycine SDS sample buffer (Invitrogen) and 2-mercaptoethanol, then boiled at 99°C for 5 min. Protein samples were separated on precast 4%–20% Novex Wedgewell Tris-glycine gels (Invitrogen) at 150 V for 1 h and 15 min. Polyvinylidene difluoride (PVDF) membranes (Millipore Sigma) were activated in 100% methanol for 1 min, then soaked in 1× transfer buffer (Invitrogen) with 10% methanol between two pieces of filter paper (ThermoFisher Scientific). The separated protein samples were transferred to PVDF membranes using a Mini Trans-Blot Electrophoretic Transfer Cell (Bio-Rad) at 100 V for 1 h. Membranes were subsequently blocked for 1 h with a 5% milk powder solution in 1× TBST. Primary antibodies were applied in 5% milk and membranes were incubated overnight at 4°C with rocking. A NARS1 antibody (Abcam, ab129162) was used at 1:1,000 dilution and the PGK1 antibody (Abcam, ab113687) was used at a 1:3,000 dilution. The following day, membranes were washed three times with 1× TBST. Secondary antibodies against mouse (for the PGK1 primary antibody; Licor, 925-68070) and rabbit (for the NARS1 primary antibody; Licor, 926-32211) were diluted in a 5% milk powder solution at a concentration of 1:20,000, along with 0.1% Tween 20 and 0.02% SDS. This solution was applied to the membranes and incubated for 1 h at room temperature with rocking. The membranes were then washed three times with 1× TBST before analyzing on a Licor Odyssey CLx Imaging System. The mean gray density of bands was quantified using the Licor Odyssey CLx Imaging System. The mean gray density of NARS1 bands was normalized to the mean gray density of their respective PGK1 band. At least three replicates were performed for each protein lysate.

### Mammalian cell culture and dimerization assays

The Nanobit MCS Starter System (Promega) was used to determine the dimerization capacity of each NARS1 protein variant. The *NARS1* open reading frame was amplified from human cDNA using primers containing *Xho*I and *Eco*RI restriction sites designed to directionally clone *NARS1* into pBiT1.1-C [TK/LgBiT], pBiT2.1-C [TK/SmBiT], pBiT1.1-N [TK/LgBiT], and pBiT2.1-N [TK/SmBiT] vectors. Gel-purified PCR products and the above pBiT plasmids were digested with *Xho*I and *Eco*RI for 3 h at 37°C, followed by heat inactivation at 65°C for 20 min. Digested pBiT vectors were then dephosphorylated with Antarctic phosphatase for 3 h at 37°C to prevent ligation of the digested plasmid DNA, followed by heat inactivation at 80°C for 2 min. The digested PCR products and dephosphorylated pBiT vectors were purified using Zymo’s Clean and Concentrator Kit. Purified products were ligated with Quick Ligase (New England Biolabs) for 15 min at room temperature and transformed into bacteria. *NARS1* variants were introduced into the wild-type *NARS1* N-SmBiT vector using site-directed mutagenesis (Agilent QuikChange II XL Site-Directed Mutagenesis Kit); primer sequences available upon request. All expression clones were validated via Sanger and nanopore sequencing.

To evaluate interactions between mutant NARS1 proteins and wild-type NARS1, HEK293T cells were transiently transfected with wild-type *NARS1* C-LgBiT, wild-type or mutant *NARS1* N-SmBiT, and a pGL4.54[luc2/TK] vector (Promega) to control for transfection efficiency and cell viability. The pGL4.54[luc2/TK] plasmid contains an HSV-TK promoter to constitutively express the reporter gene *luc2*, coding for firefly luciferase. HEK293T cells were plated (35,000 cells/well) onto a 96-well white flat-bottom plate (Corning, catalog no. 3917) and transfected using Lipofectamine 3000 (Invitrogen). After 24 h, cells were assayed using the Nano-Glo Dual-Luciferase Reporter Assay System (Promega, no. N1610). Briefly, cells were treated with ONE-Glo EX Reagent, lysed, and firefly luminescence was read using a GloMax Multi luminometer (Promega). Cells were then treated with NanoDLR Stop & Glo Reagent to quench the firefly signal and provide optimal activity for NanoLuc signal. The cells were placed on an orbital shaker at 600 rpm for 3 min, followed by resting for 7 min, and NanoLuc luminescence was read. To account for transfection efficiency, the relative luminescence units (RLUs) from NanoLuc readings were divided by the RLUs from firefly readings. All readings were then normalized to wild-type/wild-type data. Twenty-four independent transfects per construct combination were performed.

### Statistical data analysis

All statistical analyses were conducted using GraphPad Prism. To assess statistical significance, one-way ANOVA with multiple comparison tests were utilized.

## Results

### Pathogenic *NARS1* variants do not impact protein levels when expressed in yeast cells

**Figure 2:**
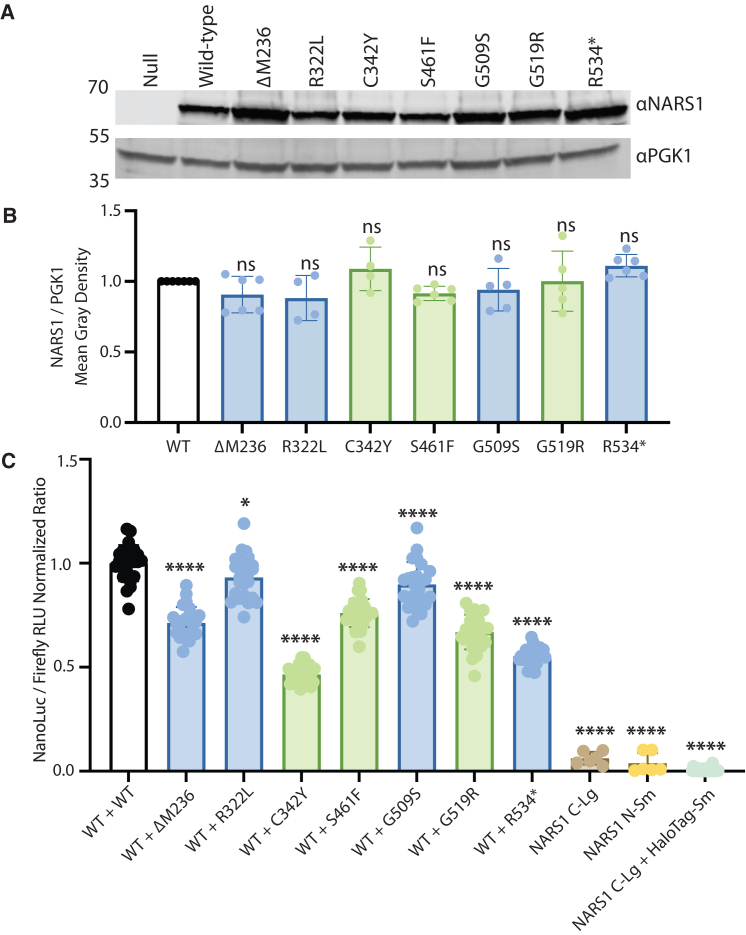
*NARS1* variants do not ablate protein expression or dimerization (A) Yeast protein lysates were subjected to western blot assays to detect human NARS1 proteins expressed from wild-type and mutant constructs, as indicated along the top. (B) Quantification of NARS1 protein expression. Green corresponds to variants associated with peripheral neuropathy and blue corresponds to variants associated with a peripheral and central nervous system phenotype. One-way ANOVA with Dunnett’s multiple comparison testing was used to determine statistical significance. (C) NanoLuc luciferase activity was expressed relative to firefly luciferase activity and then normalized to wild-type/wild-type activity. HEK293T cells expressing wild-type NARS1 C-LgBiT and HaloTag-SmBiT, as well as wild-type NARS1 C-LgBiT and wild-type NARS1 N-SmBiT independently, were used as controls. The normalized ratio is depicted on the y axis. Green corresponds to variants associated with peripheral neuropathy and blue corresponds to variants associated with a peripheral and central nervous system phenotype. One-way ANOVA with Dunnett’s multiple comparison testing was used to determine statistical significance. ∗*p* = 0.015, ∗∗∗∗*p* < 0.0001.

Previous studies revealed that ΔM236, C342Y, S461F, and G519R *NARS1* display loss-of-function effects in yeast complementation assays.^8^^,^^9^ In addition, *in vitro* aminoacylation assays demonstrated that R534∗ *NARS1* reduces enzymatic activity.^10^ These loss-of-function effects could reflect decreased protein levels (e.g., due to destabilizing missense mutations) or impaired tRNA charging. To differentiate between these two possibilities, we performed western blot analyses to assess the effects of *NARS1* variants on protein levels when expressed in yeast cells. We cloned ΔM236, R322L, C342Y, S461F, G509S, G519R, and R534∗ into the 2-μm, high-copy number, galactose-inducible pAG425 vector, transformed it into the ptetO7-*DED81* yeast strain,^39^ and isolated protein lysates. As a negative control, we designed a premature stop codon, 143-154∗ (null *NARS1*), which does not lead to detectable *NARS1* protein expression via western blot assays (Figures 2A and S1). Each pathogenic NARS1 protein variant was detected at levels comparable with wild-type NARS1, as indicated by NARS1 protein signal (Figures 2A and S1), and none of the NARS1 proteins displayed significantly decreased or ablated levels compared with wild-type NARS1 (Figures 2B and S1). Overall, these data indicate that any reduced yeast cell growth associated with the studied *NARS1* variants is not due to reduced protein levels.

### Pathogenic *NARS1* variants do not ablate the ability to dimerize with wild-type NARS1

An alternative explanation for previous reports of decreased gene function associated with pathogenic *NARS1* variants is a reduced capacity of the mutant protein to dimerize; NARS1 functions as an obligate homodimer.^40^ To test this notion, we determined if NARS1 protein variants are capable of interacting with wild-type NARS1 using the Promega NanoLuc Binary Technology (NanoBiT) protein-protein interaction system. This is a structural complementation luciferase reporter system containing a Large BiT (LgBiT) subunit and a Small BiT (SmBiT) subunit. Testing the *NARS1* truncation variant (R534∗) fused to LgBiT or SmBiT requires an N-terminal tag and since the *NARS1* C-LgBiT/*NARS1* N-SmBiT generated significant and consistent luminescent signal (Figure S2), we performed all experiments with this combination. To assess if *NARS1* variants reduce or ablate dimerization, we performed mutagenesis to introduce *NARS1* variants (ΔM236, R322L, C342Y, S461F, G509S, G519R, and R534∗) into the *NARS1* N-SmBiT vector. HEK293T cells were transfected with: (1) wild-type *NARS1* C-LgBiT, (2) mutant or wild-type *NARS1* N-SmBiT, and (3) the pGL4.54[luc2/TK] control vector. Firefly luciferase and NanoLuc luciferase activity was observed in all transfected cells (Figure S3).

All *NARS1* variants expressed in frame with an N-terminal SmBiT and co-transfected with wild-type *NARS1* expressed in frame with a C-terminal Lg-BiT displayed a positive and significant luminescent signal (Figures 2C and S3), indicating an interaction between wild-type NARS1 and mutant NARS1. Interestingly, all *NARS1* variants (ΔM236, R322L, C342Y, S461F, G509S, G519R, and R534∗) displayed a significant reduction in luminescent signal when co-transfected with wild-type *NARS1*, compared with the wild-type/wild-type combination (Figure 2C); however, these reductions do not correlate with dominant-negative effects (see below) and the introduced tags my result in steric hindrance that prevents detection of mutation-specific effects on dimerization. These findings indicate that *NARS1* variants significantly reduce, but do not ablate interactions with the wild-type NARS1 protein. In summary, in the experimental models employed here, pathogenic *NARS1* variants do not ablate protein levels or protein dimerization; both of these observations are consistent with the potential to exert a dominant-negative effect.

### Pathogenic *NARS1* variants demonstrate loss-of-function effects in yeast complementation assays

**Figure 3:**
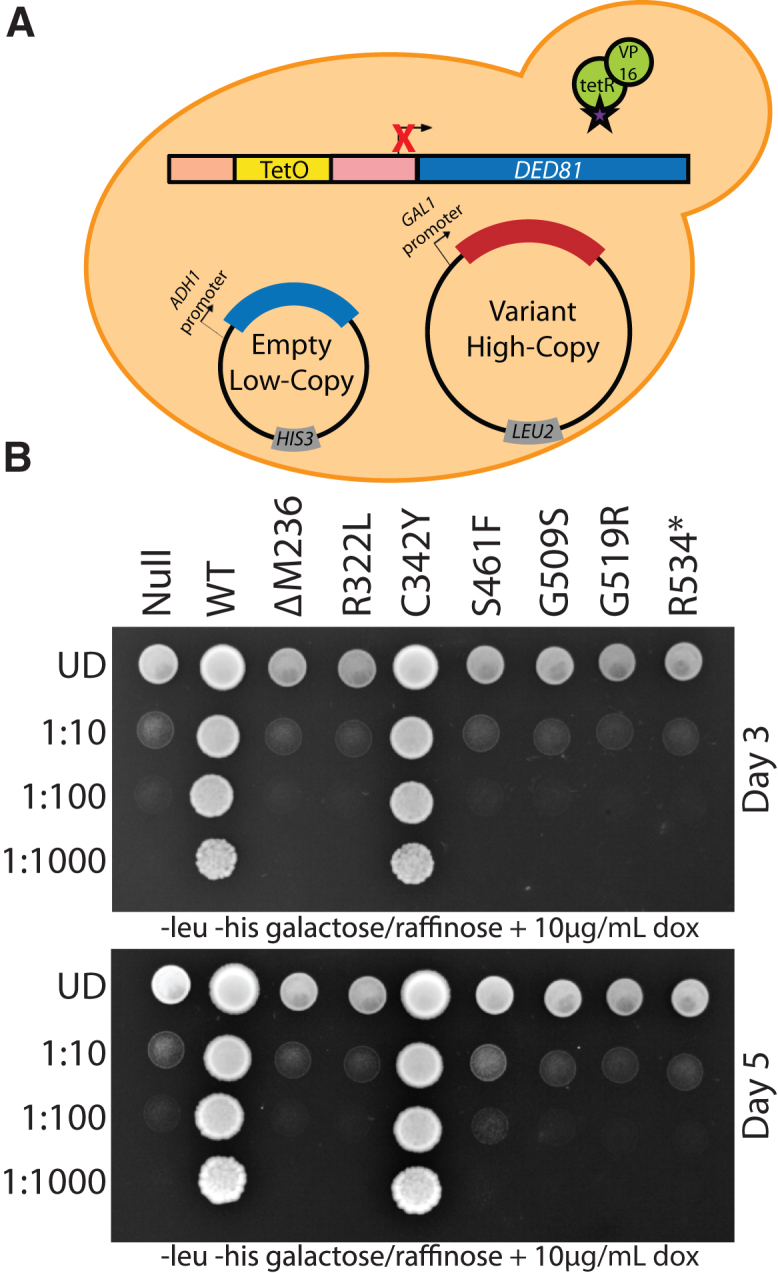
*NARS1* variants are associated with reduced yeast cell growth (A) Yeast containing a doxycycline-repressible element upstream of the endogenous *DED81* locus (the yeast ortholog of *NARS1*) were co-transformed with an empty, low-copy p413 vector and either wild-type or mutant *NARS1* in a high-copy pAG425 vector. (B) Resulting cultures were plated on galactose and raffinose media lacking leucine and histidine, and including 10 μg/mL of doxycycline. *NARS1* variants are indicated across the top and serial dilutions are noted on the left. Images were taken after 3 days of growth (top) and after 5 days of growth (bottom).

To assess the capacity of each pathogenic *NARS1* variant to independently support cellular growth, we developed a humanized yeast model system (similar to that developed for *AARS1*^27^) to express each human *NARS1* variant in the haploid ptetO7-*DED81* yeast strain (Figure 3A). This strain has *DED81* (the yeast ortholog of *NARS1*) under the control of a doxycycline-repressible promoter. We co-transformed this strain with: (1) a low-copy, centromere-bearing vector (p413) with no *NARS1* insert (empty) and (2) wild-type or mutant *NARS1* (null, ΔM236, R322L, C342Y, S461F, G509S, G519R, and R534∗) on a galactose-inducible, high-copy, 2u-bearing vector (pAG425). To test the ability of wild-type or mutant human *NARS1* to complement the loss of *DED81*, yeast cells were plated on media containing doxycycline (to repress the expression of *DED81*) and galactose (to induce expression of wild-type or mutant *NARS1* on the pAG425 vector).

When the above strains were plated on control media (i.e., without doxycycline and containing glucose or galactose) there were no observable growth defects (Figure S4). When plated on media containing both galactose and doxycycline, a *NARS1* null allele was unable to support yeast cell growth, consistent with *DED81* being an essential gene and with the complete repression of *DED81* on solid growth medium (Figures 3B and S5). As previously reported previously,^8^^,^^9^ wild-type human *NARS1* displayed robust yeast cell growth, indicating that human *NARS1* can complement the loss of *DED81* (Figure 3B). In contrast, ΔM236, R322L, S461F, G509S, G519R, and R534∗ did not support yeast cell growth (Figure 3B), indicating that they are loss-of-function mutations in this assay. Interestingly, after 5 days of growth, S461F *NARS1* exhibited yeast cell growth, but reduced compared with wild-type *NARS1* (Figure 3B), indicating that it is a hypomorphic allele in this assay. In contrast, C342Y *NARS1*, a previously reported hypomorphic allele,^8^ exhibited similar yeast cell growth compared with wild-type *NARS1* (Figure 3B). In summary, with the exception of C342Y *NARS1*, all pathogenic *NARS1* variants studied demonstrate loss-of-function effects in yeast complementation assays, consistent with the potential to exert a dominant-negative effect.

### Pathogenic *NARS1* alleles repress yeast cell growth when co-expressed with wild-type *NARS1*

**Figure 4:**
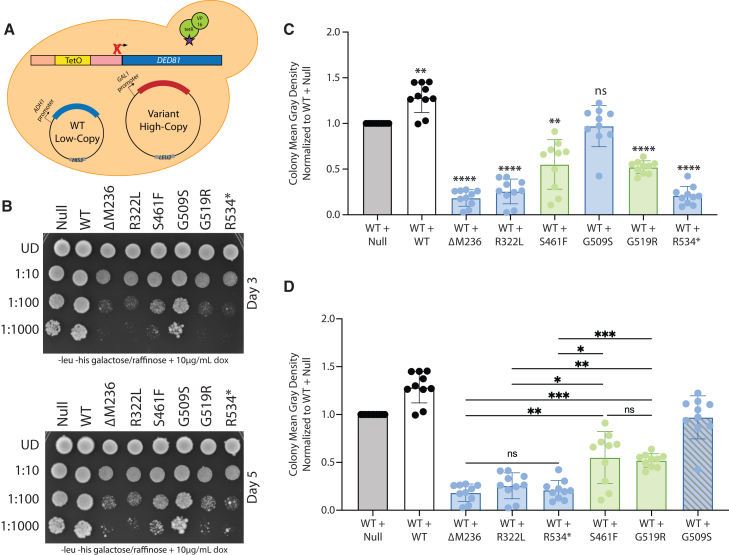
Pathogenic *NARS1* variants cause dominant growth defects in yeast when co-expressed with wild-type *NARS1* (A) Yeast containing a doxycycline-repressible element upstream of *DED81* (the yeast ortholog of *NARS1*) were co-transformed with wild-type *NARS1* in the p413 vector and either wild-type or mutant *NARS1* in the pAG425 vector. (B) Resulting cultures were plated on galactose and raffinose media lacking leucine and histidine, with 10 μg/mL of doxycycline. *NARS1* variants are indicated across the top and serial dilutions are noted on the left. Images were taken after 3 days of growth (top) and after 5 days of growth (bottom). (C) Yeast spot intensity at the 1:100 dilution was measured using ImageJ analysis and then normalized to the “WT + Null” sample. Error bars indicate the standard deviation. To test for statistically significant differences in yeast growth between each strain and the WT + Null strain, a one-way ANOVA with Dunnett’s multiple comparisons test was used. ∗∗*p* = 0.002, ∗∗∗∗*p* < 0.001. (D) Normalized mean gray density as described in (C). To test for statistically significant differences among all strains, a one-way ANOVA with Tukey’s multiple comparisons test was used. Given that G509S does not exert a dominant-negative effect, samples were not compared with this allele (indicated by a striped blue bar). ∗*p* = 0.01, ∗∗*p* = 0.007, ∗∗∗*p* = 0.0001.

To test for dominant-negative properties of loss-of-function *NARS1* variants, we transformed ΔM236, R322L, S461F, G509S, G519R, and R534∗ *NARS1* in pAG425 into the ptetO7-*DED81* yeast strain harboring wild-type *NARS1* on p413 (Figure 4A); C342Y *NARS1* would not be expected to have dominant-negative properties in the described yeast model since it displayed function similar to wild-type *NARS1* in the current study (see discussion) and was therefore not studied in this assay. Null and wild-type *NARS1* were also transformed into this yeast strain as controls. When spotted on media containing galactose and doxycycline, strains co-expressing wild-type *NARS1* and ΔM236, R322L, S461F, G519R, or R534∗ *NARS1* exhibited significantly reduced yeast cell growth when compared with strains co-expressing wild-type *NARS1* and a *NARS1* null allele or wild-type *NARS1* (Figures 4B and 4C). That is, the expression of the above five variants significantly decreased yeast cell growth even though the yeast cells had sufficient wild-type *NARS1* for robust growth; these data suggest that the mutant alleles produce proteins that bind to and repress the function of wild-type NARS1 proteins. In contrast, G509S *NARS1* displayed robust yeast cell growth when co-expressed with wild-type *NARS1* (Figures 4B and 4C), suggesting that this allele does not have dominant-negative properties (see discussion). In an attempt to quantify the observed dominant-negative effects of *NARS1* variants when co-expressed with wild-type *NARS1*, we performed growth curves in liquid media containing galactose and 200 μg/mL of doxycycline. Consistent with our spotting data, ΔM236, R322L, S461F, G519R, and R534∗ caused significantly reduced yeast cell growth when co-expressed with wild-type *NARS1* (Figure S6A); however, quantitation of the dominant-negative effects was complicated by incomplete *DED81* repression in liquid media (Figure S6B).

**Figure 5:**
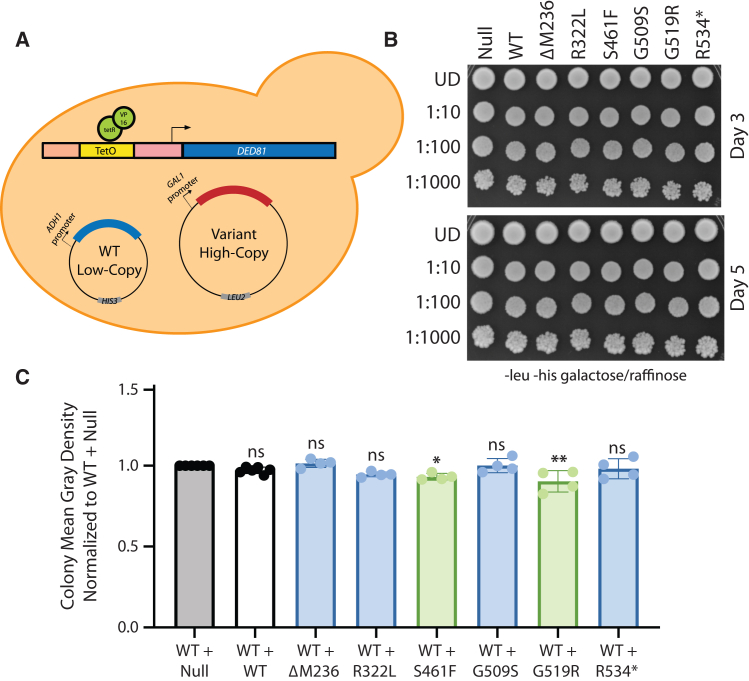
*DED81* rescues the dominant growth phenotype caused by pathogenic *NARS1* variants (A) Yeast containing a doxycycline-repressible element upstream of *DED81* (the yeast ortholog of *NARS1*) were co-transformed with wild-type *NARS1* in the p413 vector and either wild-type or mutant *NARS1* in the pAG425 vector. (B) Resulting cultures were plated on galactose and raffinose media lacking leucine and histidine, and with no doxycycline. *NARS1* variants are indicated along the top and serial dilutions are noted on the left. Images were taken after 3 days of growth (top) and after 5 days of growth (bottom). (C) Yeast spot intensity at the 1:100 dilution was measured using ImageJ analysis and then normalized to the “WT + Null” sample. Error bars indicate the standard deviation. To test for statistically significant differences in yeast growth between each strain and the WT + Null strain, a one-way ANOVA with Dunnett’s multiple comparisons test was used. ∗*p* = 0.0356, ∗∗*p* = 0.0018.

One possible explanation for the observed clinical heterogeneity of dominant neurological diseases caused by *NARS1* variants (Figure 1B) is that variable dominant-negative effects may temper (PNS only) or exacerbate (PNS + CNS involvement) clinical outcomes. To begin to explore this, we analyzed the yeast growth data in the context of the clinical phenotypes. As noted above, with the exception of G509S, all *NARS1* variants repressed yeast cell growth when co-expressed with wild-type *NARS1* (Figures 4B and 4C). Interestingly, the PNS + CNS alleles (blue shading in Figure 4D) had a more severe effect on yeast cell growth compared with the PNS alleles (green shading in Figure 4D); there were no significant differences between alleles within each group (Figure 4D). Finally, re-establishing *DED81* expression by plating on media with galactose and without doxycycline (Figure 5A) rescues the growth phenotype observed when *NARS1* variants are co-expressed with wild-type *NARS1* (Figures 5B, 5C, and S7). Combined, these data indicate that the yeast growth defect associated with pathogenic *NARS1* variants is due to the repression of asparaginyl-tRNA synthetase function and suggest that a more severe reduction in this function (i.e., through a more severe dominant-negative effect) may result in disease features beyond the PNS.

**Figure 6:**
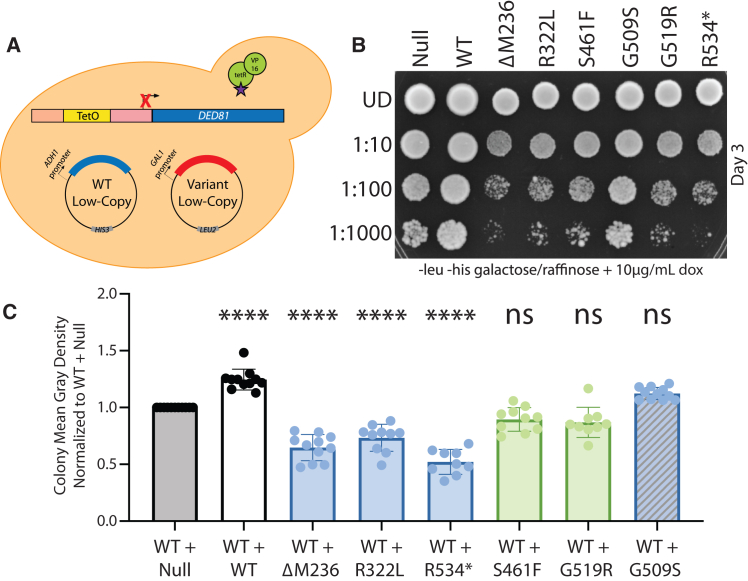
*NARS1* variants retain dominant growth defects when expressed from a low-copy number vector (A) Yeast containing a doxycycline-repressible element upstream of *DED81* (the yeast ortholog of *NARS1*) harboring wild-type *NARS1* in the p413 vector were co-transformed with either wild-type or mutant *NARS1* in the pAG415 vector. (B) Resulting cultures were plated on galactose and raffinose media lacking leucine and histidine, and containing 10 μg/mL of doxycycline. *NARS1* variants are indicated along the top and serial dilutions are noted on the left. Images were taken after 3 days of growth. (C) Yeast spot intensity at the 1:100 dilution was measured using ImageJ analysis and then normalized to the “WT + Null” sample. Error bars indicate the standard deviation. To test for statistically significant differences in yeast growth between each strain and the WT + Null strain, a one-way ANOVA with Dunnett’s multiple comparisons test was used. ∗∗∗∗*p* < 0.001.

A limitation of the above-mentioned dominant yeast assay is the imbalance in allelic expression, where the pathogenic allele is expressed from a high-copy vector and the wild-type allele is expressed from a low-copy vector; this design was intentional to increase the chances of detecting a dominant-negative effect in yeast.^27^ To address this limitation, we repeated the above study using two low-copy vectors (Figure 6A). We cloned ΔM236, R322L, S461F, G509S, G519R, R534∗, and wild-type *NARS1* into pAG415 and confirmed the loss-of-function effects of pathogenic variants in this vector (Figure S8). Next, we transformed each of the above mutant *NARS1* constructs into the ptetO7-*DED81* yeast strain harboring wild-type *NARS1* on p413. The pAG415 vector contains a *GAL1* promoter to induce expression of *NARS1* in the presence of galactose, a centromeric origin of replication to yield a low-copy vector number per cell, and a *LEU2* auxotrophic marker. Null and wild-type *NARS1* in pAG415 were also transformed into the yeast strain as controls. Consistent with our findings above, ΔM236, R322L, and R534∗ *NARS1* cause a reduction in yeast cell growth when co-expressed with wild-type *NARS1* (Figures 6B, 6C, and S11); however, S461F and G519R *NARS1* no longer display a significant repression in yeast cell growth in this new expression system. Importantly, while the p413 and pAG415 vectors both contain a centromeric origin of replication and are expected to result in similar copy number, the pAG415 vector has at least a 10-fold increase in expression compared with the p413 vector (Figures S9 and S10); thus, this experimental system does not fully address the above-mentioned limitation. Interestingly, the clinically relevant differences between *NARS1* alleles is again observed as the three variants that retain a significant dominant growth defect in this experiment are associated with both PNS and CNS phenotypes.

In sum, our data demonstrate dominant-negative properties of pathogenic *NARS1* variants that cause dominant neurological diseases. This is the second report of dominant-negative effects for neuropathy-associated ARS loci, suggesting that this may be a unifying mechanism for the seven ARS loci implicated in this phenotype.

## Discussion

Here, we present data indicating that pathogenic asparaginyl-tRNA synthetase (*NARS1*) variants that cause dominant neurological phenotypes have dominant-negative properties. We developed a humanized yeast assay and demonstrated that six out of seven pathogenic *NARS1* variants do not support yeast cell growth, indicating that they are loss-of-function alleles. Additionally, we used yeast and mammalian experimental systems to show that *NARS1* variants do not grossly affect protein levels nor do they ablate the ability to dimerize with wild-type NARS1. To test for a dominant-negative effect, we co-expressed loss-of-function *NARS1* alleles with wild-type *NARS1* and showed that the pathogenic variants impair the ability of wild-type *NARS1* to support yeast cell growth; reactivation of the endogenous yeast gene (*DED81*) rescued this defect, directly demonstrating that the growth phenotype is caused by impaired asparaginyl-tRNA synthetase function. Finally, the dominant reduction of yeast cell growth on solid media varied among *NARS1* variants as follows: (1) subtle defects were observed for neuropathy (PNS)-associated variants, which correlates well with the milder symptoms in these patients^8^^,^^9^ and (2) more severe defects were observed for variants associated with both PNS and CNS phenotypes. Therefore, this study advances our understanding of the pathogenic mechanism of dominant diseases caused by mutations in genes encoding ARSs and provides initial insights into genotype-phenotype correlations.

While our studies demonstrated that *NARS1* variants do not ablate interactions between wild-type and mutant NARS1 proteins, interactions were significantly reduced between mutant and wild-type proteins. The observed reductions do not correlate with dominant-negative effects, which raises questions on the interpretation of the former data. There are two important limitations to the dimerization assay that we performed. First, the use of the LgBiT tag may introduce steric hindrance, which impacts dimer formation, making it difficult to observe mutation-specific effects on dimerization; however, it is worth noting that if such steric hindrance effects were significant, a similar reduction in dimerization would be expected for the wild-type NARS1 homodimer. Second, these experiments were conducted using HEK293T cells, which do not capture the physiological context of *NARS1*-mediated neurological disease. Additional studies utilizing neurons would be beneficial to test for neuron-specific effects on expression or dimerization toward implicating dominant-negative effects in *NARS1*-mediated neurological disease.

One of the *NARS1* variants studied here (C342Y) did not demonstrate loss-of-function effects in the assay developed in this study; however, we previously showed that this variant is a hypomorphic allele in a traditional 5-FOA-based yeast complementation model.^8^ Similarly, another *NARS1* variant studied here (S461F) demonstrated a hypomorphic effect in this study, while it was previously shown to be a complete loss-of-function allele.^8^ These discrepancies are likely due to the use of an *ADH1* promoter in the prior study compared with the use of a *GAL1* promoter in this study. Studies have shown that the *GAL1* promoter directs higher expression of genes in a galactose-dependent manner, compared with the *ADH1* promoter.^41^ Additional analyses are necessary to assess if gene expression varies between our current and previous yeast models. While hypomorphic alleles have been shown to have dominant-negative potential,^42^ testing this for C342Y *NARS1* is beyond the resolution of the experiments described in this study and will require quantitative biochemical analyses that assess both the mutant/mutant and mutant/wild-type NARS1 dimer.^43^

A third variant studied here (G509S *NARS1*) showed loss-of-function effects but did not demonstrate dominant growth defects and was therefore not deemed to be a dominant-negative allele in the yeast assay that we developed. We believe that there are two possible explanations for this discrepancy. First, the pathogenicity of this variant should be revisited as G509S *NARS1* is a *de novo* variant identified in only one family via whole-exome sequencing.^10^ This may be a rare, benign variant, or the patient carrying G509S may carry a loss-of-function, non-coding *NARS1* variant on the other allele; recessive *NARS1*-related disease is similar to certain cases of dominant *NARS1*-related disease.^10^ Second, G509S *NARS1* may have functional consequences not assessed in the yeast assay presented in this study, which tests for functional consequences in the *NARS1* open reading frame. For example, the human splicing finder, a bioinformatics application designed to predict splicing signals,^44^ identifies that G509S *NARS1* potentially activates a cryptic splice acceptor site. Since this variant maps to the last exon of *NARS1*, this altered splicing may result in a truncated protein similar to R534∗, which did show dominant-negative properties in our studies. Mini-gene splicing assays or analyzing transcripts from patient cells will be required to explore this latter possibility.

Although the data presented here suggests that *NARS1* variants function through a dominant-negative mechanism, additional studies are required to determine if the interaction between mutant NARS1 and wild-type NARS1 is suppressing the function of the wild-type protein. One way to address this is to impair the ability of mutant NARS1 to interact with wild-type NARS1. We have shown that introducing a dimer-reducing variant in *cis* with pathogenic alanyl-tRNA synthetase (*AARS1*) variants prevents them from interacting with wild-type AARS1 and rescues dominant-negative effects in yeast.^27^ Similar to how restoring *DED81* expression alleviated the dominant growth defect associated with pathogenic *NARS1* alleles in yeast, it will be important to determine if over-expressing wild-type *NARS1* rescues the dominant phenotype in animal models. In addition, over-expression of tRNA-gly in mutant *GARS1* fly and mouse models rescues peripheral neuropathy phenotypes.^26^ Similarly, studies on patient fibroblast cells with bi-allelic variants in *IARS1*, *LARS1*, *FARSB*, and *SARS1* have shown that increasing the dose of the respective amino acid increases aminoacylation activity *in vitro*. In the same study, patients were prescribed an oral treatment of the respective amino acid and all presented with an improved phenotype.^45^ Thus, it will be important to test if over-expression of tRNA-asn and/or asparagine supplementation can rescue the dominant *NARS1*-associated phenotype.

It will also be important to further investigate the differential dominant-negative effects of *NARS1* variants, as our data revealed a potential correlation between dominant growth defects and phenotypic severity. Studies to quantify the binding affinities of mutant NARS1 to wild-type NARS1 would be valuable to determine if there is differential binding that correlates with disease severity. Interestingly, four out of seven *NARS1* variants (ΔM236, R322L, G509S, and R534∗) implicated in a dominant neurological disorder share phenotypic overlap with *NARS1*-mediated recessive neurological disease. This includes global developmental delay, seizures, intellectual disability, ataxia, and neuropathy. Bi-allelic ARS variants associated with early-onset recessive disorders affect multiple tissues,^46^ and in certain instances, include a peripheral neuropathy phenotype.^11^^,^^47^^,^^48^ These observations support an impairment in enzyme activity as a common feature of all ARS-mediated inherited diseases. More importantly, these observations suggest that ARS-mediated dominant and recessive diseases exist along a spectrum, relating to the degree of reduction in ARS activity, rather than as two separate categories of disease. Thus, it would be prudent to routinely test for a neuropathy phenotype when assessing patients with ARS-related recessive disease.^11^^,^^49^^,^^50^

Overall, the findings in this study add evidence for a unifying dominant-negative mechanism for ARS-mediated dominant neurological disease. We demonstrated that variants in a second cytoplasmic, dimeric ARS implicated in dominant disease exhibit dominant-negative properties. Additionally, our data suggest that the clinical heterogeneity observed in *NARS1*-mediated dominant neurological disease may be linked to differential dominant-negative effects.

## Data and code availability

No large datasets or code were generated or analyzed in this study.

## Acknowledgments

The authors would like to thank all of the patients and families that participated in previous studies on pathogenic *NARS1* variants; Dr. Martin Arlt for helpful discussion on growth curve assays; and Dr. Phyllis Hanson, Dr. Jacob Kitzman, Dr. Lev Prasov, and Dr. Thomas Wilson for helpful discussions and suggestions. S.M.P. is supported by the Michigan Pre-doctoral Training in Genetics Program (T32GM149391). A.A. is supported by a grant from the 10.13039/100000057National Institute of General Medical Sciences (GM136441). J.B. is supported by an 10.13039/501100003130FWO Senior Clinical Researcher mandate (1805021N) and is a member of the European Reference Network for Rare Neuromuscular Diseases (ERN EURO-NMD) and the μNEURO Research Center of Excellence of the 10.13039/501100007660University of Antwerp. S.Z. is supported by the Charcot Marie Tooth (10.13039/100002028CMT) Association, the 10.13039/100005202Muscular Dystrophy Association (MDA), and 10.13039/100000002National Institutes of Health (NIH) grants 5U54NS065712 and 5R01NS105755.

## Declaration of interests

The authors declare no competing interests.

## Supplemental information

Document S1. Figures S1–S11

Document S2. Article plus supplemental information
