# Supplementary material for: Asparaginyl-tRNA synthetase (NARS1) variants implicated in dominant neurological phenotypes display dominant-negative properties
Source: HGG Adv. 2025 Sep 18;7(1):100519. doi: 10.1016/j.xhgg.2025.100519 (PMC12513288; doi:10.1016/j.xhgg.2025.100519)
Supplement: Document S1. Figures S1–S11 [file mmc1.pdf]

HGGA, Volume 7

Supplemental information

Asparaginyl-tRNA synthetase (*NARS1*) variants implicated in dominant neurological phenotypes display dominant-negative properties

Sheila M. Peeples, Keyana Blake, Brendan L.M. Sutton, Marina Konyukh, Stephan Züchner, Tanya Stojkovic, Jonathan Baets, and Anthony Antonellis

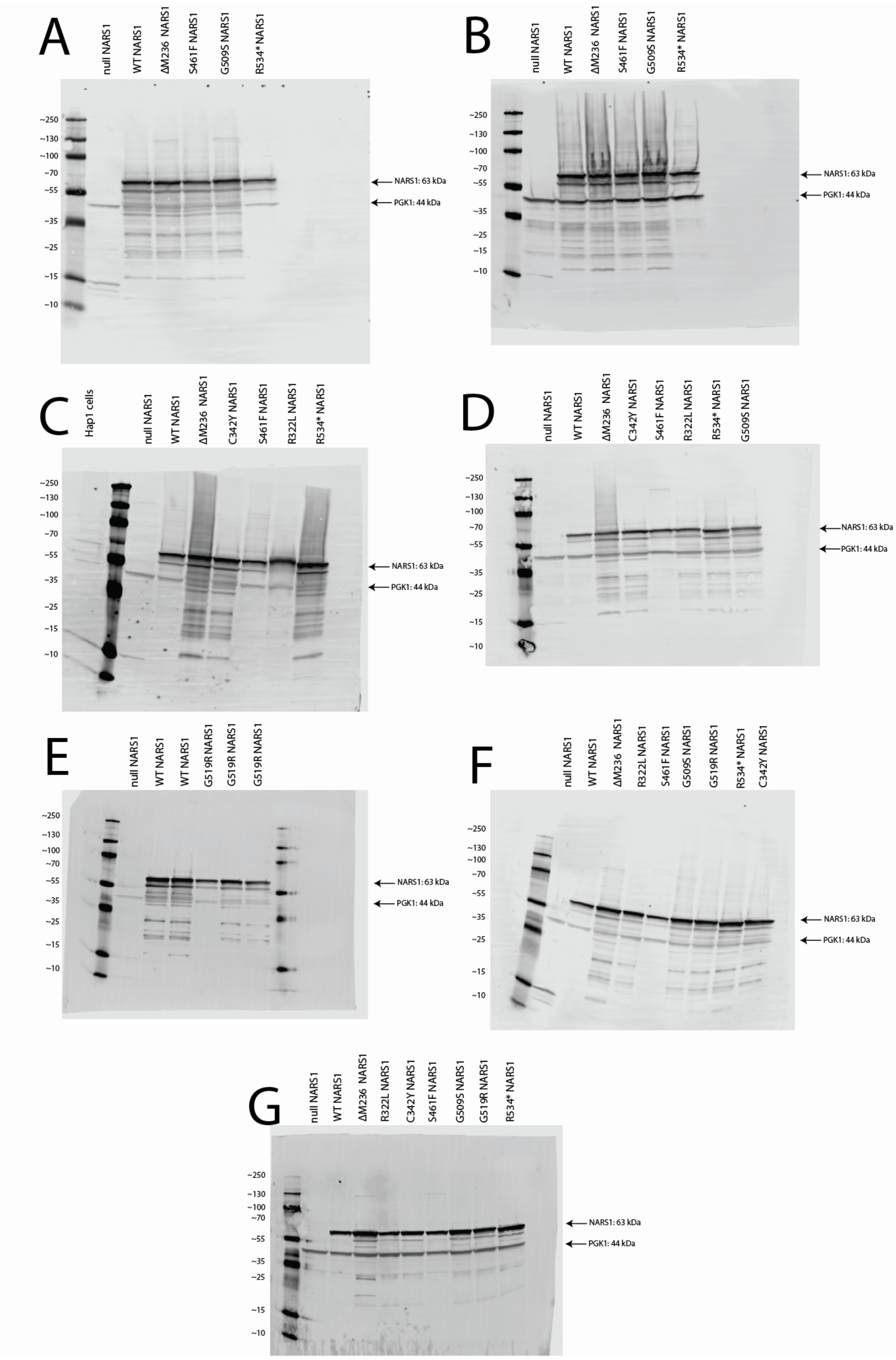

Figure S1. Uncropped western blot images corresponding to main Figure 2. All replicates are shown.

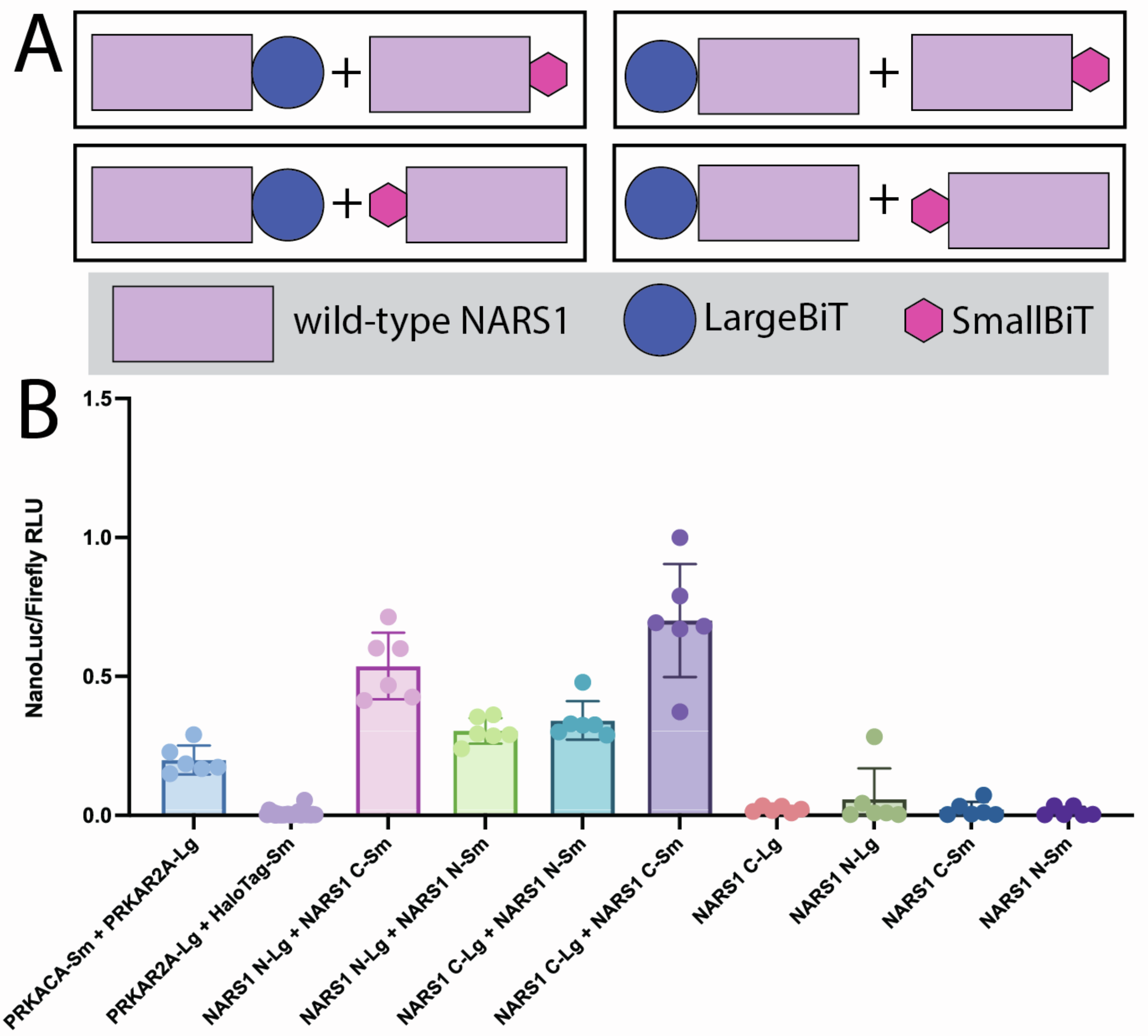

Figure S2. Screening NARS1 NanoBiT expression constructs for optimal luminescent signal. (**A**) Four combinations of *NARS1* expression constructs in-frame with either LargeBiT or SmallBiT were tested for optimal signal. Wild-type NARS1 is displayed as a purple rectangle, LargeBiT is displayed as a blue circle, and SmallBiT is depicted as a pink hexagon. (**B**) Firefly and NanoLuc luciferase activity was measured from transfected HEK293T cells using Promega's Nano-Glo dual luciferase reporter assay. The LargeBiT and SmallBiT vector combinations are noted across the bottom of the graph. PRKACA-SmBiT and PRKAR2A-LgBiT were used as a positive control, and PRKAR2A-LgBiT and HaloTag-SmBiT were used as a negative control. NanoLuc luciferase activity was normalized to firefly luciferase activity and the normalized ratio is depicted on the y-axis. Each dot represents an independent transfection for a total of 24 biological replicates.

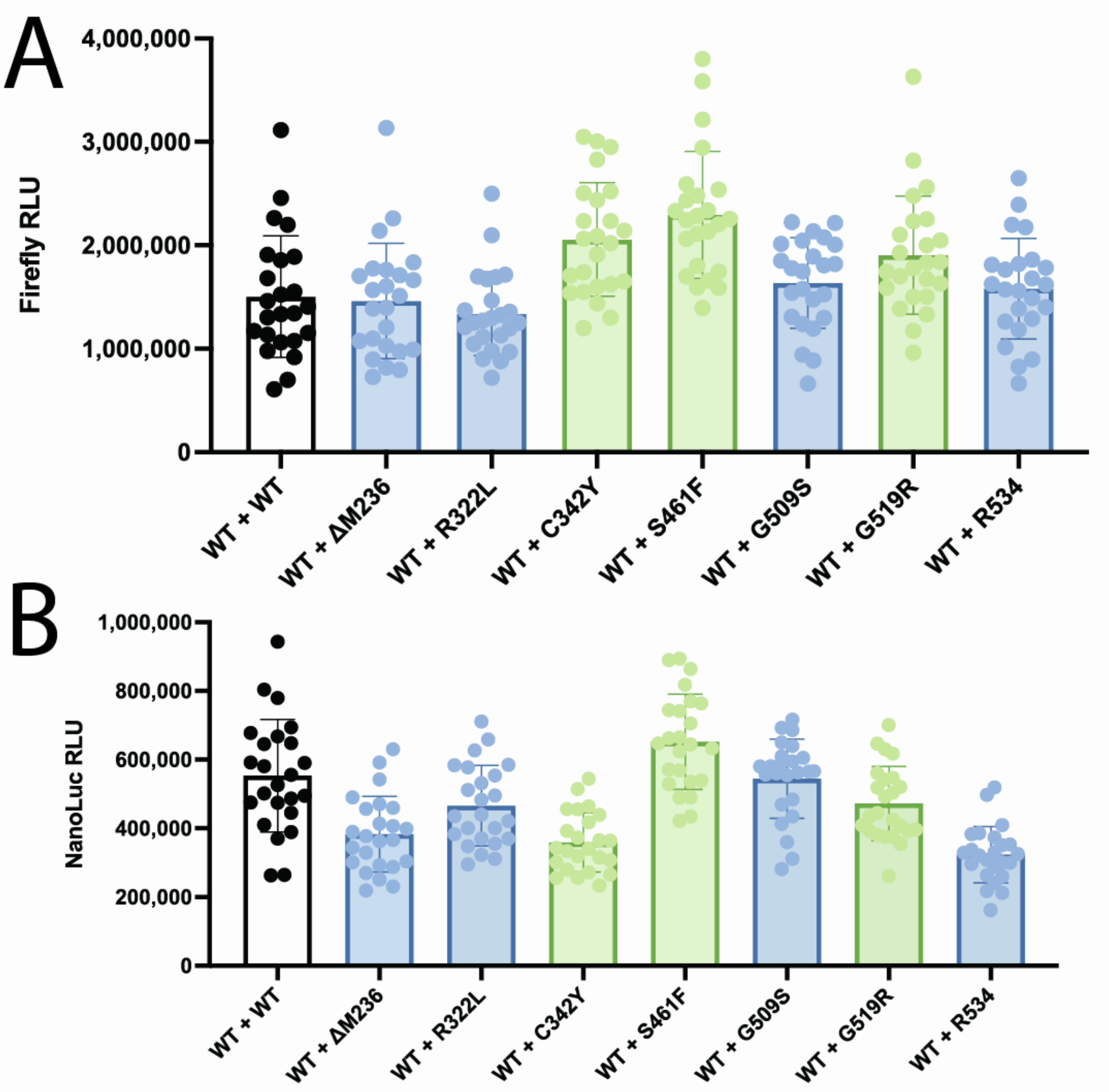

Figure S3. Raw data from firefly and NanoLuc luciferase activity experiments. (**A**) Firefly luciferase activity and (**B**) NanoLuc luciferase activity measured from HEK293T cells expressing wild-type NARS1 C-LgBiT and either wild-type or mutant NARS1 N-SmBiT. The LargeBiT and SmallBiT vector combinations are noted across the bottom of the graphs. In both panels, each dot represents an independent transfection for a total of 24 biological replicates.

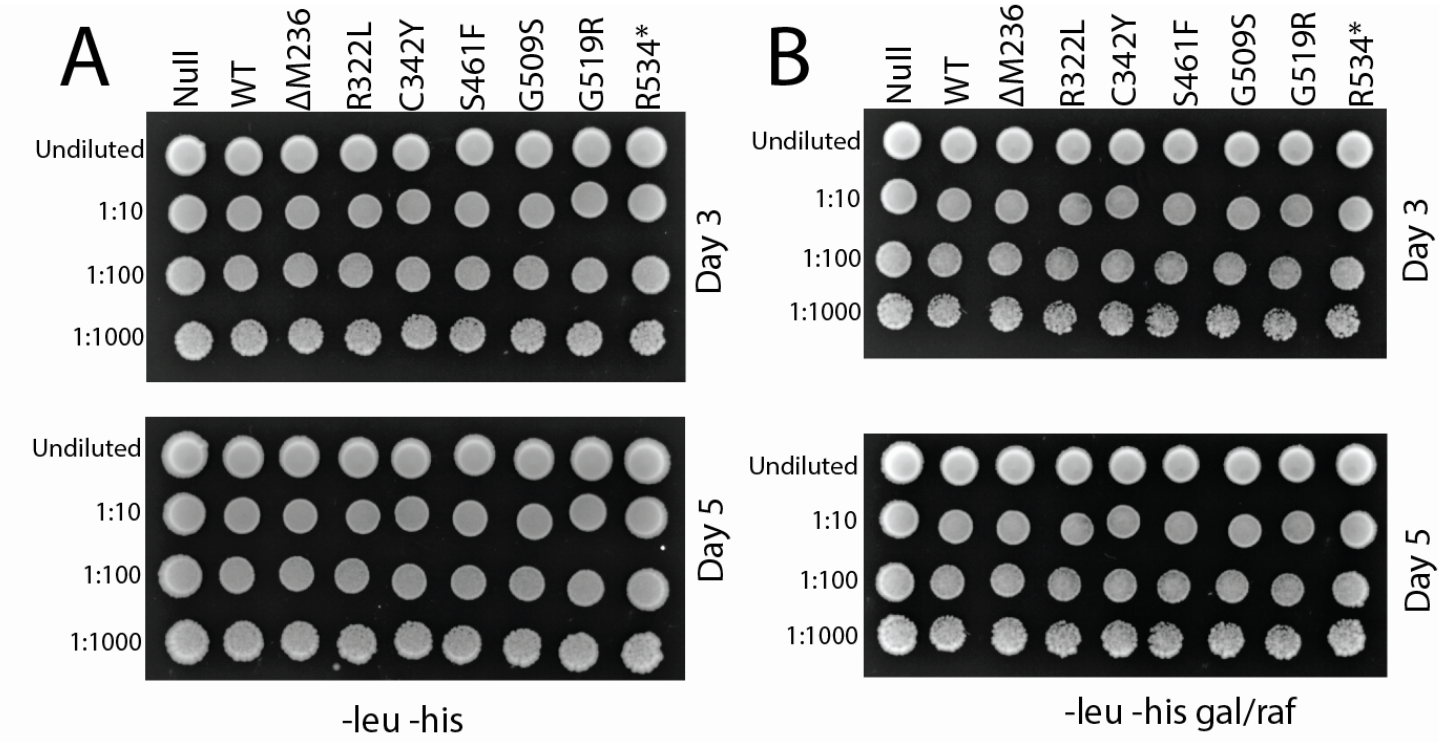

Figure S4. Yeast expressing pathogenic *NARS1* variants do not exhibit growth defects on media lacking doxycycline. Yeast containing a doxycycline repressible element upstream of endogenous *DED81* (yeast ortholog of *NARS1*) were co-transformed with an empty p413 expression vector and either wild-type or mutant *NARS1* in the pAG425 expression vector. Resulting cultures were plated on (**A**) glucose or (**B**) galactose and raffinose media lacking leucine and histidine. *NARS1* variants are indicated across the top and serial dilutions are noted on the left. Images were taken after 3 days of growth (top panels) and after 5 days of growth (bottom panels). For all panels, a representative image is shown from 3 total replicates per variant.

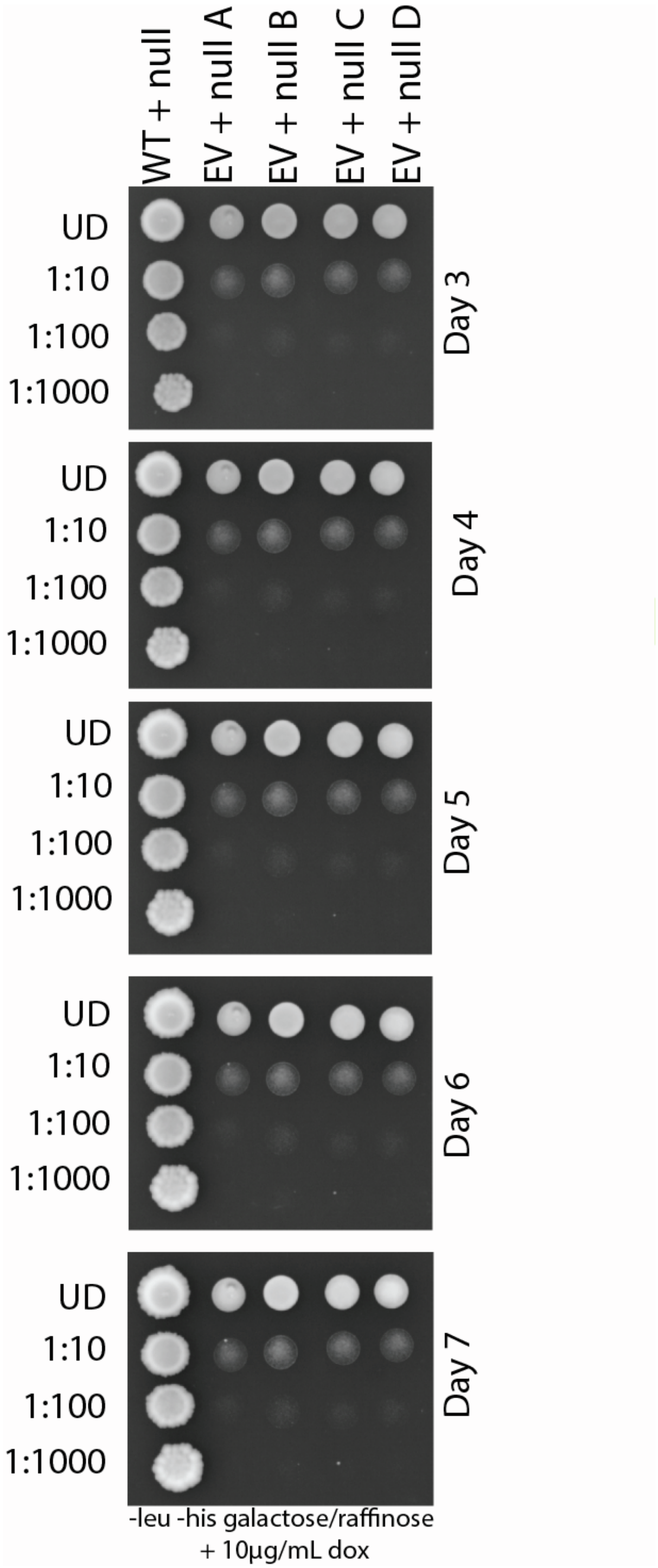

Figure S5. *DED81* is repressed on solid growth medium containing doxycycline. Yeast containing a doxycycline repressible element upstream of the endogenous *DED81* locus were co-transformed with an empty, low-copy p413 vector and either wild-type or mutant *NARS1* in a high-copy pAG425 vector. Resulting cultures were plated on galactose and raffinose media lacking leucine and histidine, and including 10µg/mL of doxycycline. *NARS1* variants are indicated across the top and serial dilutions are noted on the left. Images were obtained after 3-7 days of growth indicated on the right.

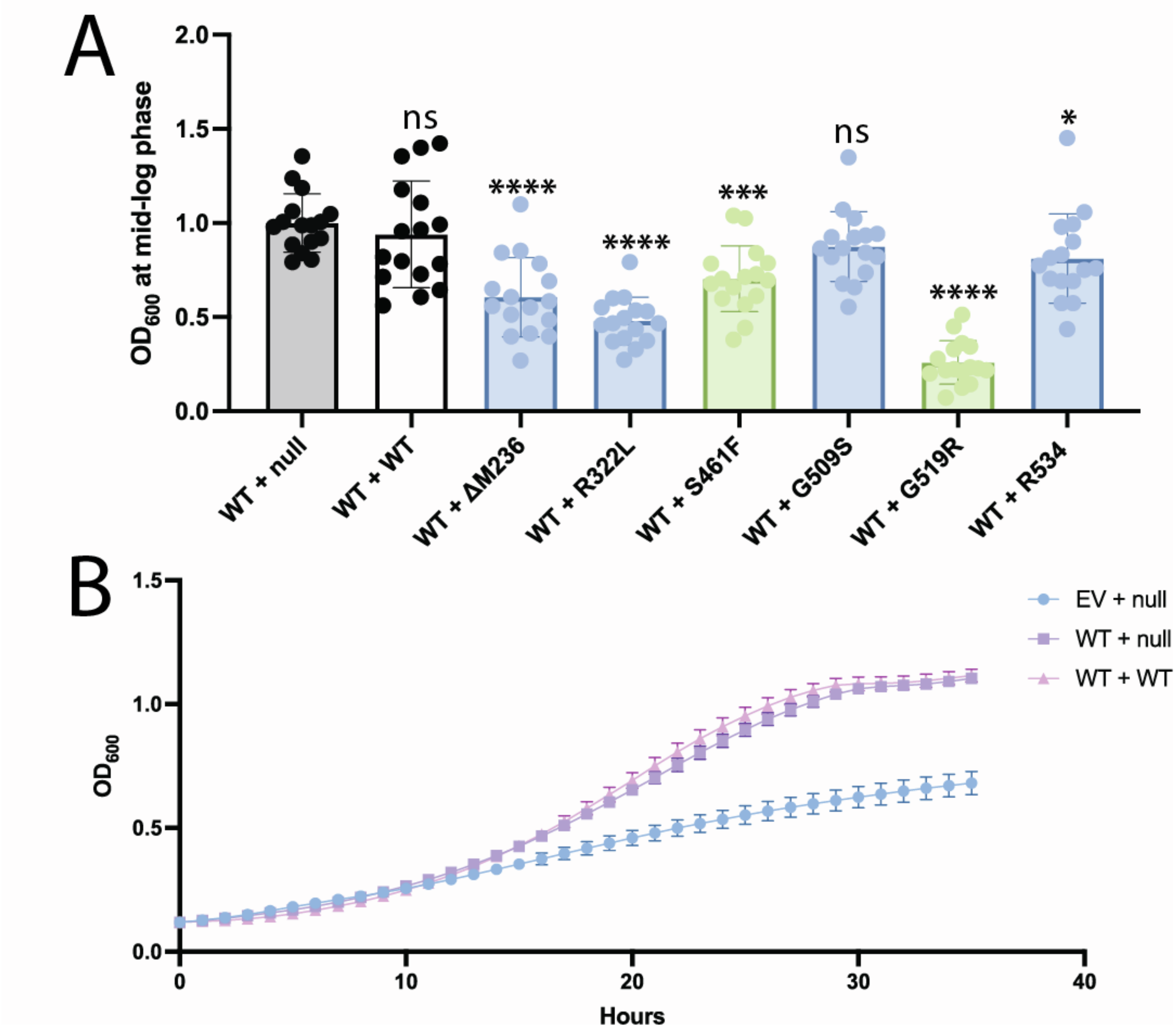

Figure S6. Growth curve analysis of the ptet-*DED81* yeast strain in the presence of doxycycline to repress *DED81*. Yeast containing a doxycycline repressible element upstream of endogenous *DED81* (yeast ortholog of *NARS1*) were co-transformed with a wild-type *NARS1* p413 expression vector and either wild-type or mutant *NARS1* in the pAG425 expression vector. Yeast were grown in liquid galactose and raffinose media lacking leucine and histidine, with 200µg/mL of doxycycline and OD_{600} readings were measured every hour for 36 hours. (**A**) To compare yeast growth to that of the strain expressing both wild-type and null *NARS1*, the OD_{600} at mid-log phase was plotted and a one-way ANOVA with Dunnett's multiple comparisons test was used. (**B**) OD_{600} readings measured every hour for 36 hours. Note the significant growth associated with the sample with no human *NARS1* ("EV + null") indicating incomplete repression of *DED81* in liquid culture. In both panels, each dot represents a biological replicate for a total of 16 replicates.

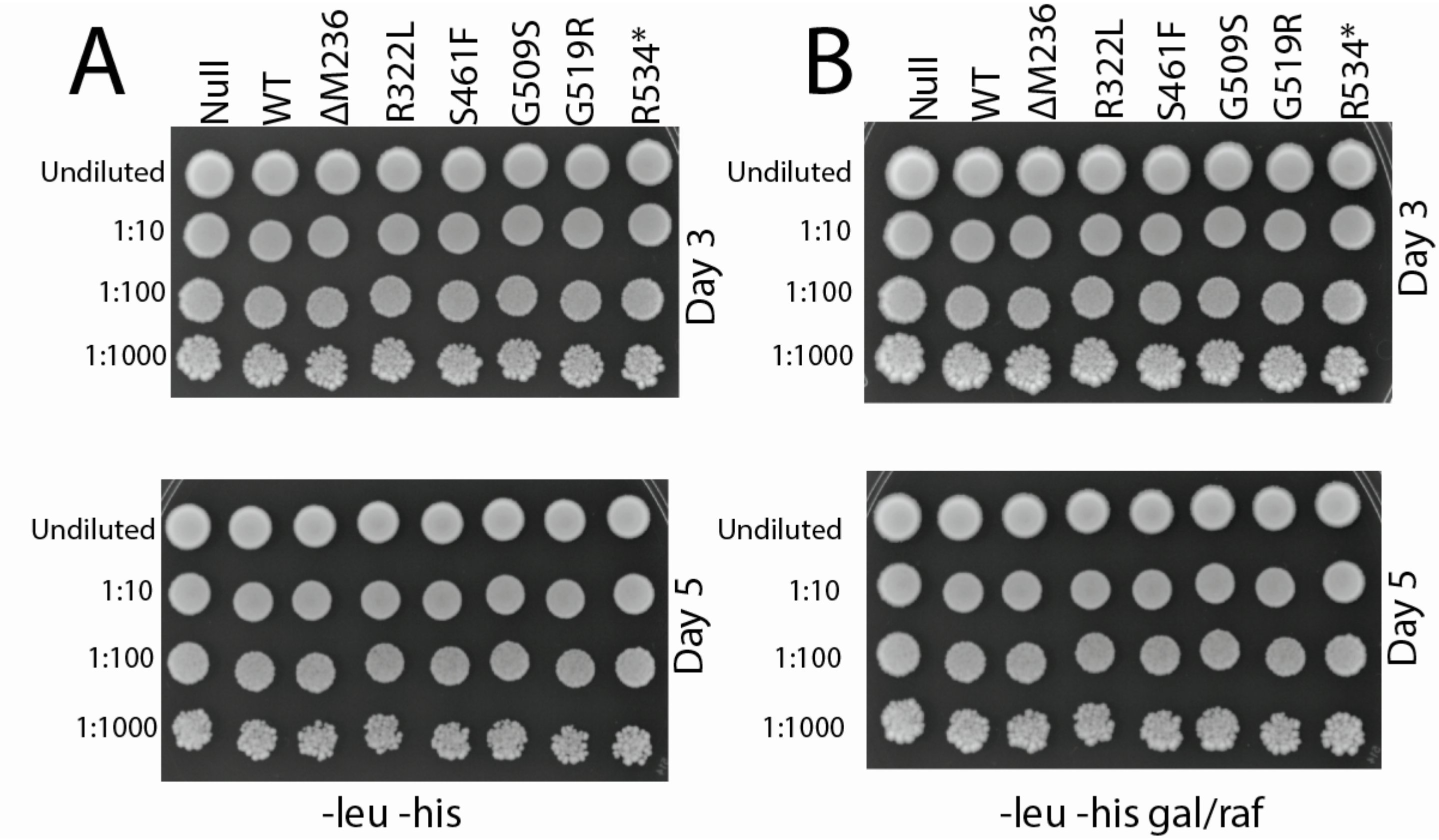

Figure S7. Yeast co-expressing WT *NARS1* and pathogenic *NARS1* variants do not exhibit growth defects on media lacking doxycycline. Yeast containing a doxycycline repressible element upstream of endogenous *DED81* (yeast ortholog of *NARS1*) were co-transformed with a wild-type *NARS1* p413 expression vector and either wild-type or mutant *NARS1* in the pAG425 expression vector. Resulting cultures were plated on (**A**) glucose or (**B**) galactose and raffinose media lacking leucine and histidine. *NARS1* variants are labeled across the top and serial dilutions are noted on the left. Images were taken after 3 days of growth (top panels) and after 5 days of growth (bottom panels). For all panels, a representative image is shown from 10 total replicates per variant.

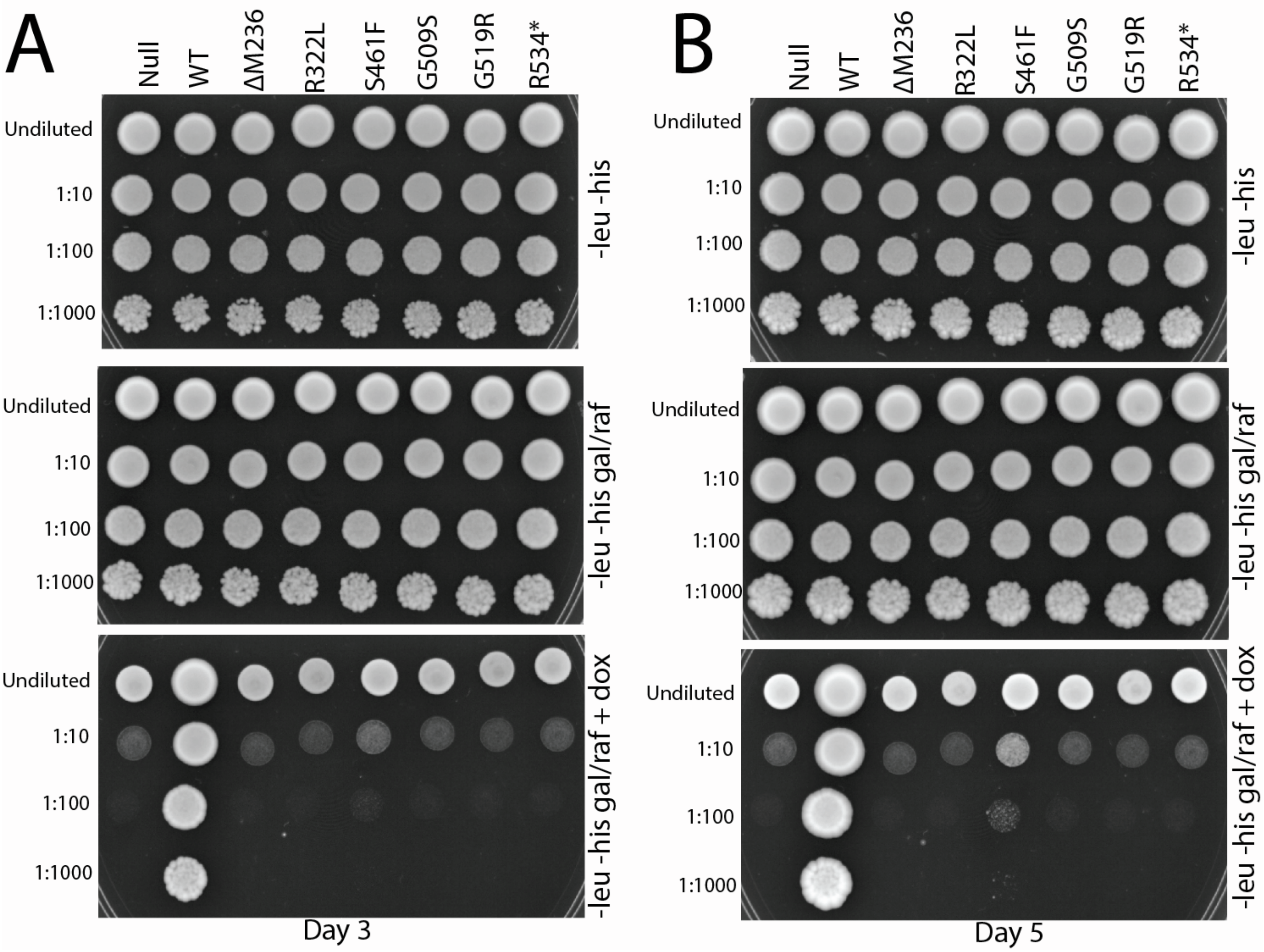

Figure S8. Pathogenic *NARS1* variants retain loss-of-function effects when expressed from a low-copy number vector. Yeast containing a doxycycline repressible element upstream of endogenous *DED81* (yeast ortholog of *NARS1*) were co-transformed with an empty p413 vector and either wild-type or mutant *NARS1* in the pAG415 expression vector. Resulting cultures were plated on either glucose media lacking leucine and histidine (top panels), galactose and raffinose media lacking leucine and histidine (middle panels), or galactose and raffinose media lacking leucine and histidine with 10µg/mL of doxycycline (bottom panels). *NARS1* variants are labeled along the top and serial dilutions are displayed on the left. Images were taken after (**A**) 3 days of growth and after (**B**) 5 days of growth. For all panels, a representative image is shown from 3 total replicates per variant.

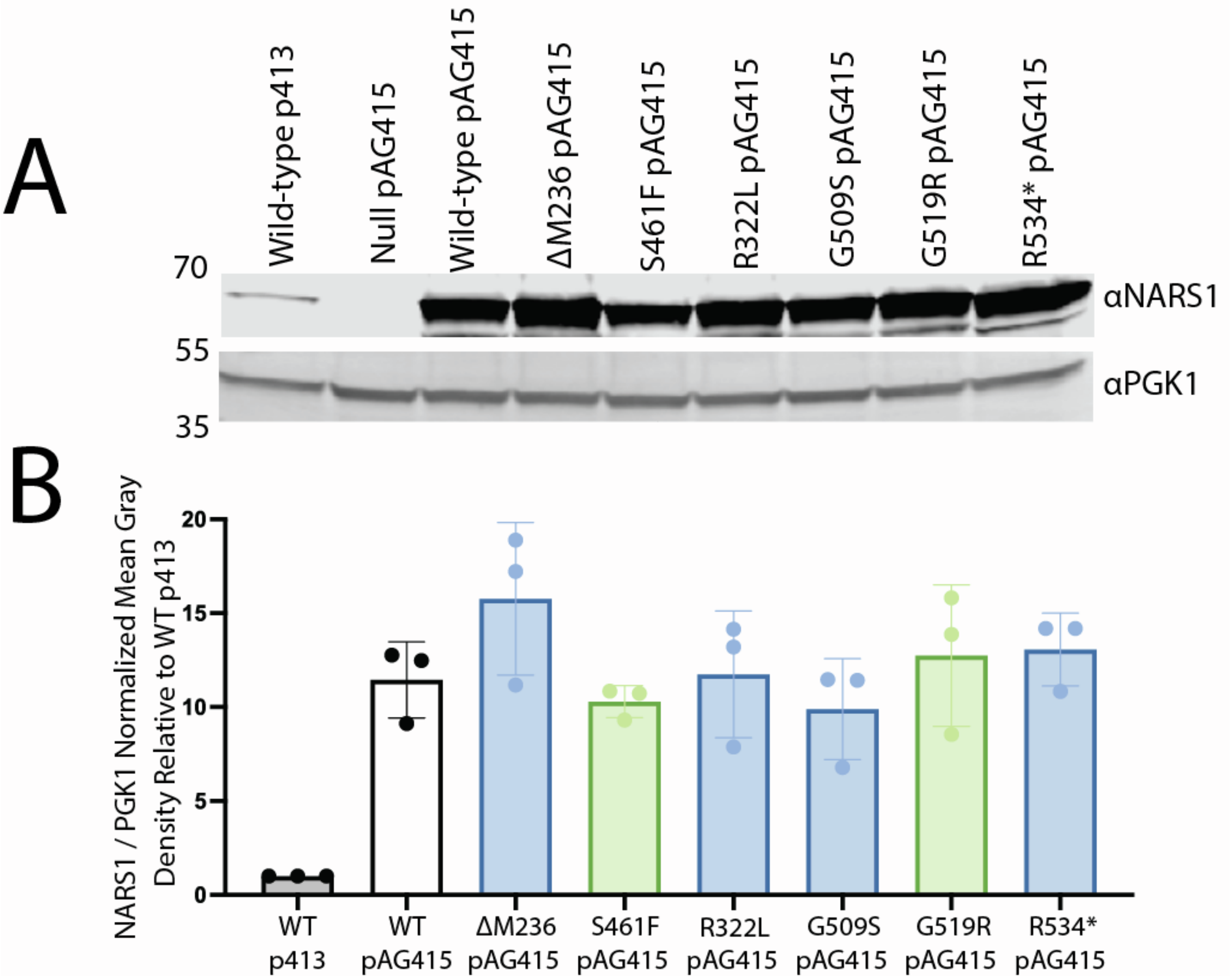

Figure S9. pAG415 has a 10-fold increase in expression compared to p413 (A)Yeast protein lysates were subjected to western blot assays to detect human NARS1 proteins expressed from wild-type and mutant constructs, as indicated along the top. (B) Quantification of NARS1 protein expression. Green corresponds to variants associated with peripheral neuropathy and blue corresponds to variants associated with a peripheral and central nervous system phenotype.

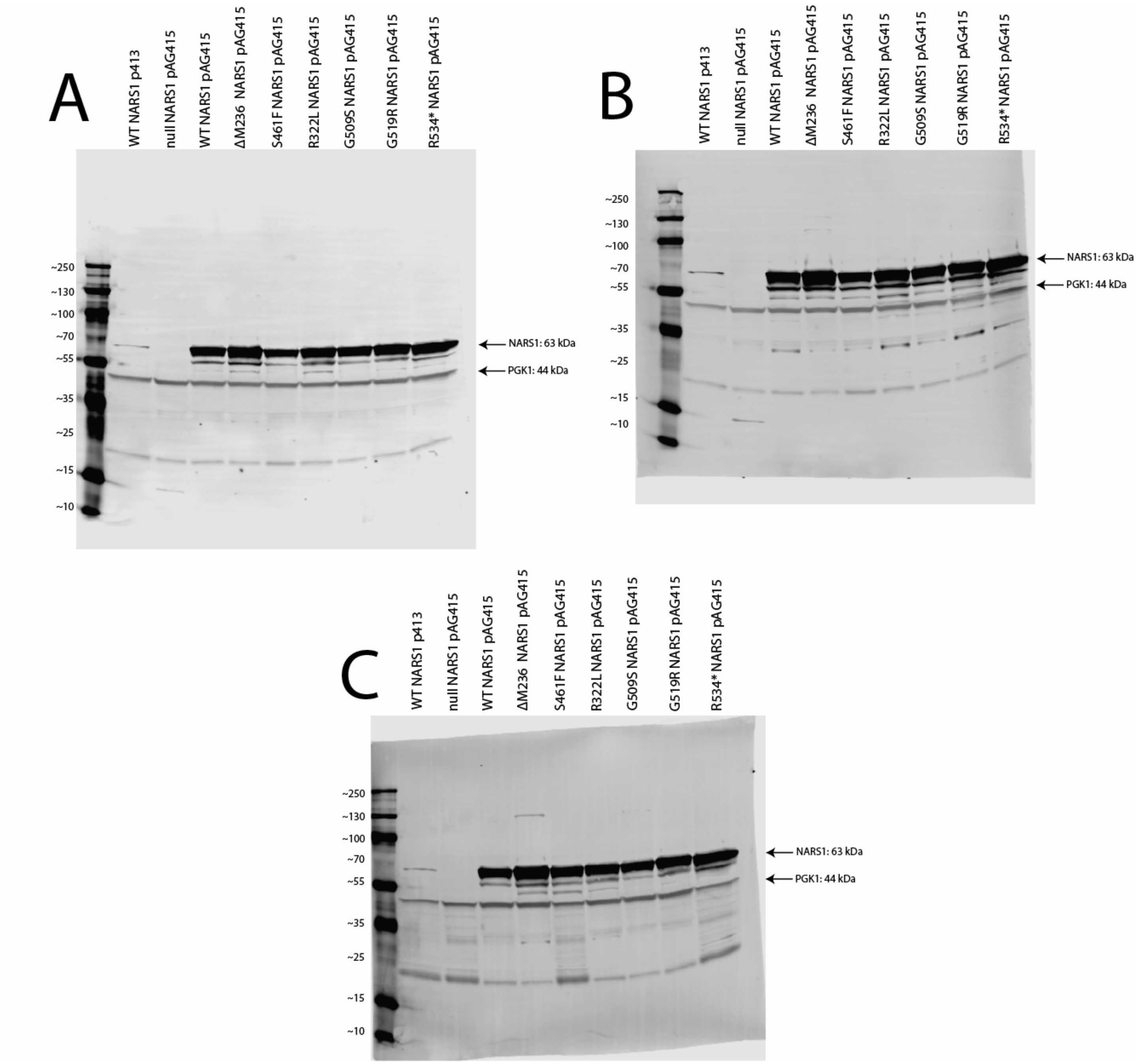

Figure S10. Uncropped western blot images corresponding to Supplemental Figure 9. All replicates are shown.

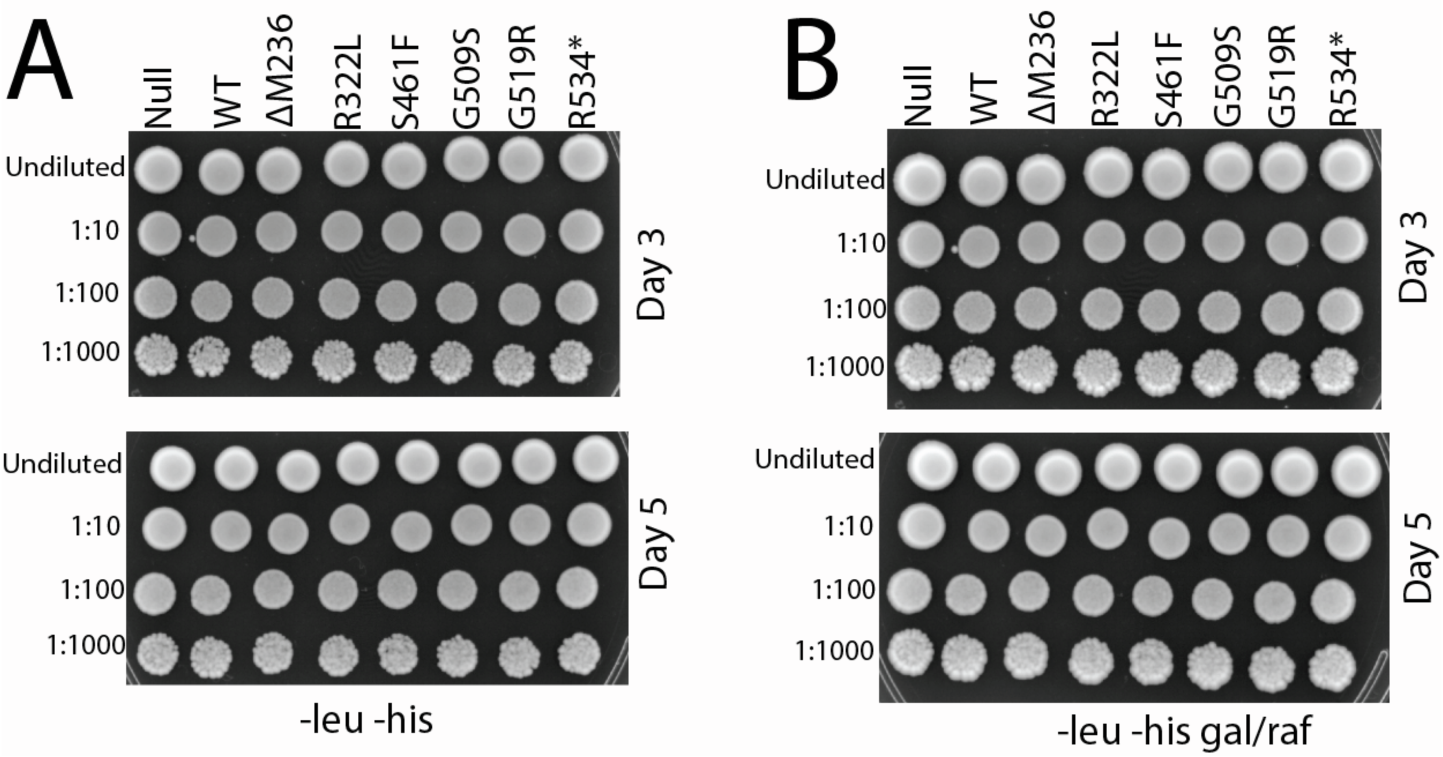

Figure S11. Yeast co-expressing WT *NARS1* and *NARS1* variants on cen-bearing vectors do not exhibit growth defects on media lacking doxycycline. Yeast containing a doxycycline repressible element upstream of endogenous *DED81* (yeast ortholog of *NARS1*) were co-transformed with a wild-type *NARS1* p413 expression vector and either wild-type or mutant *NARS1* in the pAG415 expression vector. Resulting cultures were plated on (**A**) glucose or (**B**) galactose and raffinose media lacking leucine and histidine. *NARS1* variants are labeled at the top and serial dilutions are displayed on the left. Images were taken after 3 days of growth (top) and after 5 days of growth (bottom). For all panels, a representative image is shown from 10 total replicates per variant.
